# Supplementary material for: Cell detoxification of secondary metabolites by P4-ATPase-mediated vesicle transport
Source: eLife. 2023 Jul 4;12:e79179. doi: 10.7554/eLife.79179 (PMC10322151; doi:10.7554/eLife.79179)
Supplement: Figure 1—figure supplement 2—source data 2. — M, DNA Marker DL2000; 1, PCR product of plasmid vector; 2, PCR product of BbCRPA disruption mutant (ΔBbCRPA); 3, PCR product of wide-type B.bassiana (WT); 4, PCR product of heterologous recombinant mutant. File for the primary data corresponding to Figure 1—figure supplement 2B. [file elife-79179-fig1-figsupp2-data2.zip › PCR analysis of BbCRPA disruption mutant.pptx]

## Slide 1
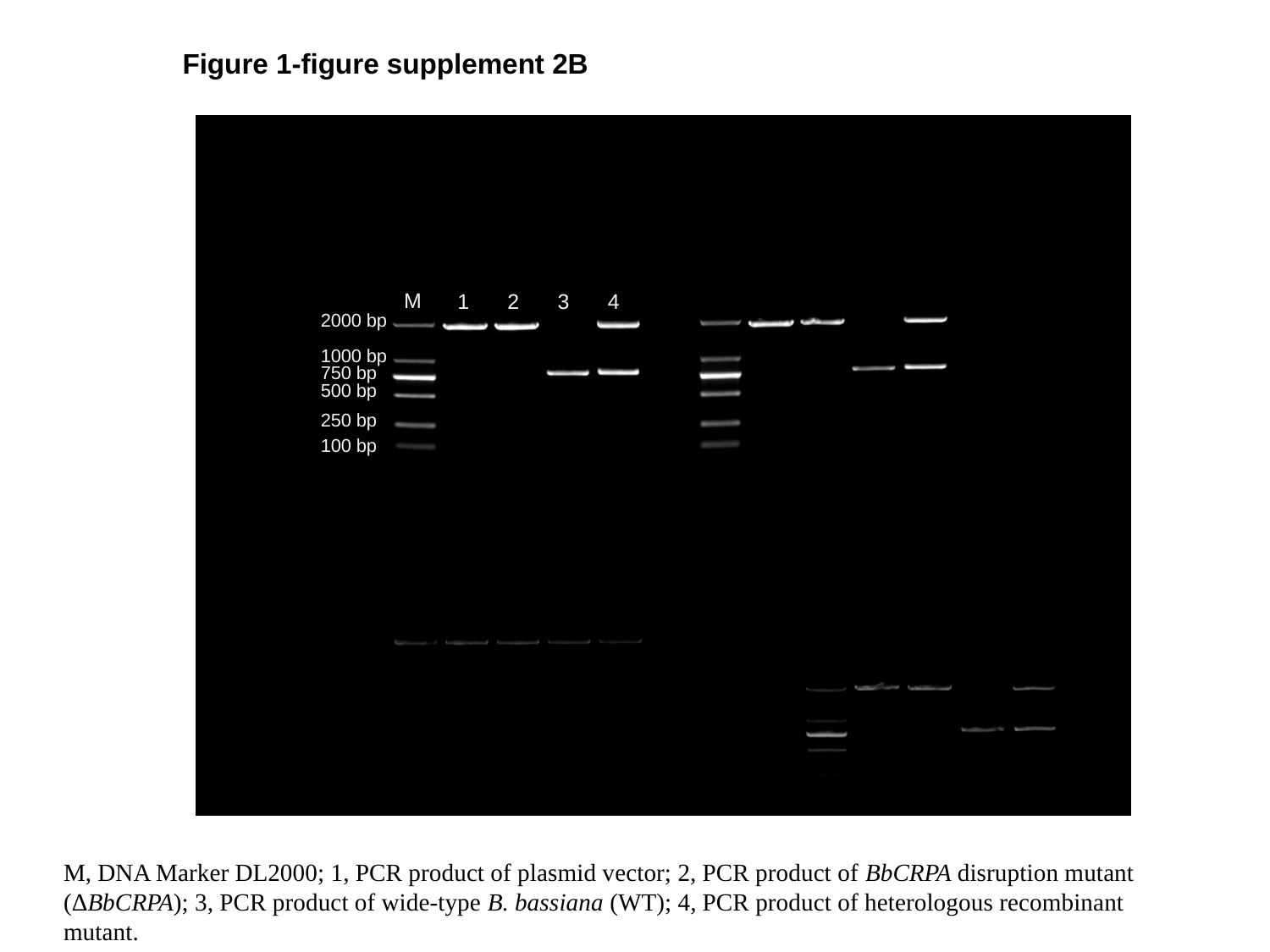

Figure 1-figure supplement 2B
M
1
2
3
4
2000 bp
1000 bp
750 bp
500 bp
250 bp
100 bp
M, DNA Marker DL2000; 1, PCR product of plasmid vector; 2, PCR product of BbCRPA disruption mutant (ΔBbCRPA); 3, PCR product of wide-type B. bassiana (WT); 4, PCR product of heterologous recombinant mutant.
