# Supplementary figures and images for: Cell detoxification of secondary metabolites by P4-ATPase-mediated vesicle transport
Source: eLife. 2023 Jul 4;12:e79179. doi: 10.7554/eLife.79179 (PMC10322151; doi:10.7554/eLife.79179)

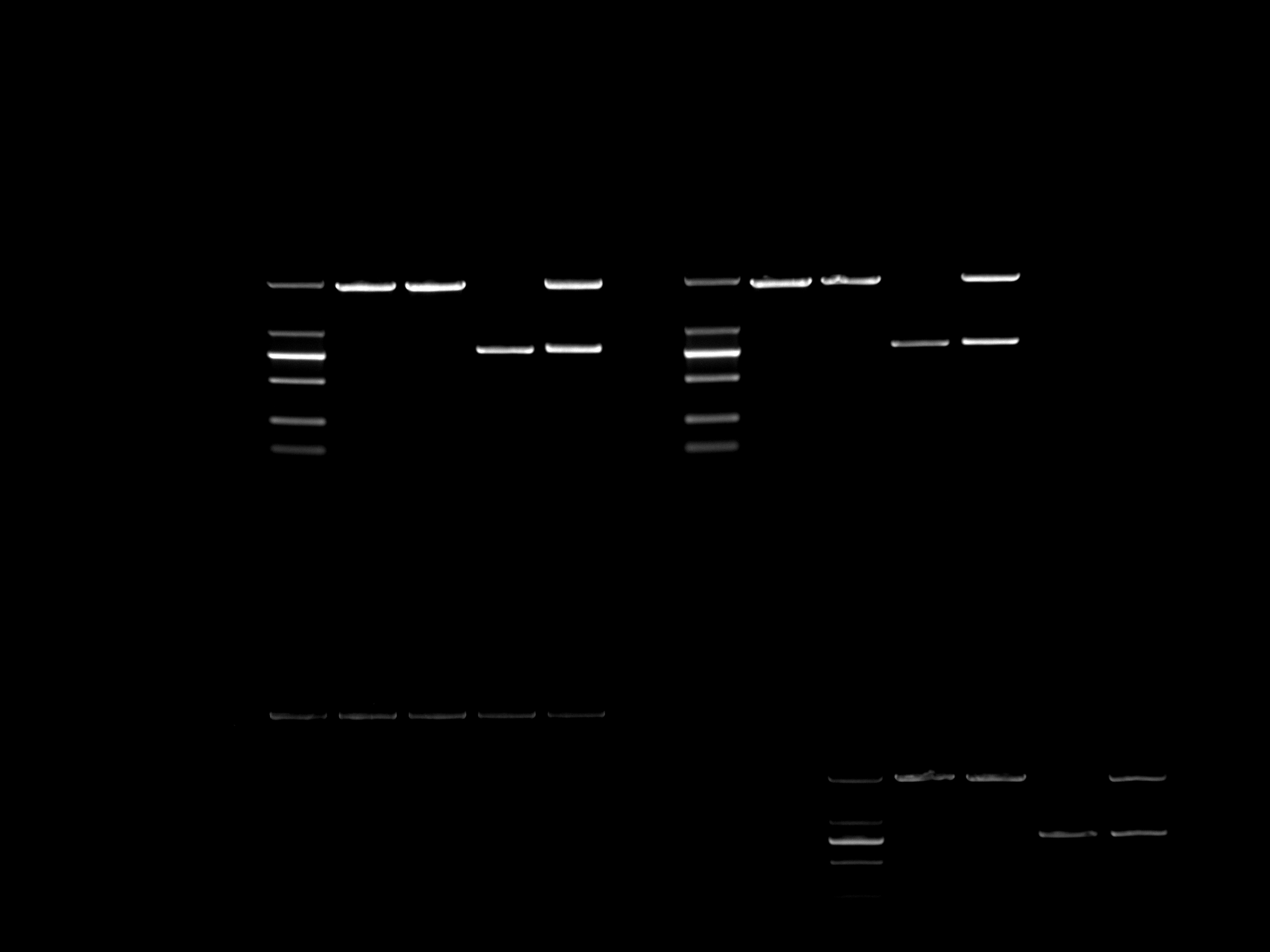

Supplement: Figure 1—figure supplement 2—source data 2. — M, DNA Marker DL2000; 1, PCR product of plasmid vector; 2, PCR product of BbCRPA disruption mutant (ΔBbCRPA); 3, PCR product of wide-type B.bassiana (WT); 4, PCR product of heterologous recombinant mutant. File for the primary data corresponding to Figure 1—figure supplement 2B. [file elife-79179-fig1-figsupp2-data2.zip › Original image-PCR analysis of BbCRPA disruption mutant.jpg]

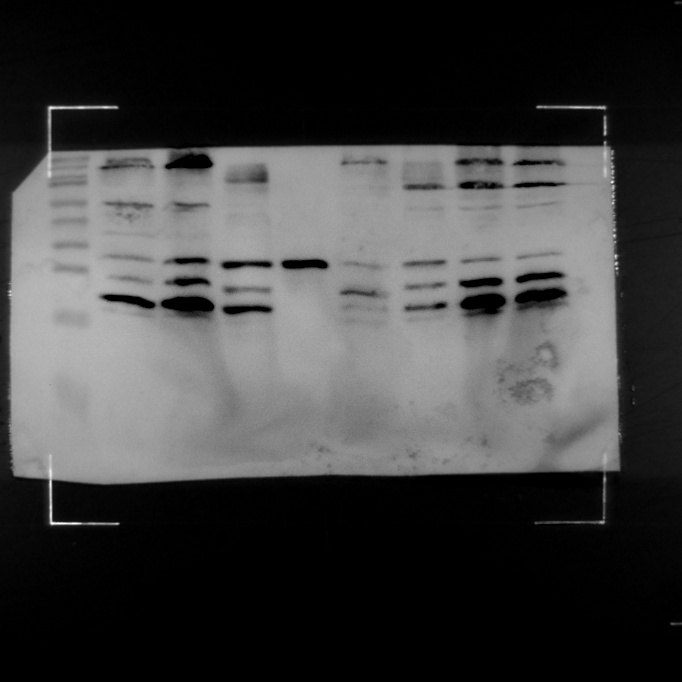

Supplement: Figure 4—figure supplement 1—source data 2. — Uncropped western blot is to demonstrate K264 in N terminus is responsible for the ubiquitination of BN258-268 (K264A)::mRFP. Hyphae were incubated with anti-mRFP affinity beads 4FF. IP materials (anti-mRFP) were subjected to immunoblot with anti-Ub (ubiquitin) and anti-mRFP antibodies. The total extracts (input) were detected by immunoblot with anti-mRFP antibody. +: CsA (20 μg/ml), -: DMSO. File for the primary data corresponding to Figure 4—figure supplement 1G. [file elife-79179-fig4-figsupp1-data2.zip › Original image-Anti-mRFP-input.jpg]

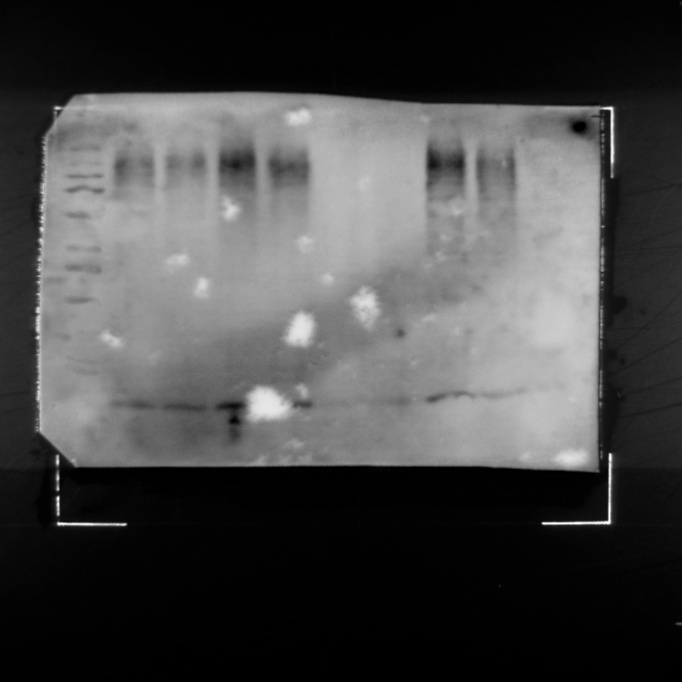

Supplement: Figure 4—figure supplement 1—source data 2. — Uncropped western blot is to demonstrate K264 in N terminus is responsible for the ubiquitination of BN258-268 (K264A)::mRFP. Hyphae were incubated with anti-mRFP affinity beads 4FF. IP materials (anti-mRFP) were subjected to immunoblot with anti-Ub (ubiquitin) and anti-mRFP antibodies. The total extracts (input) were detected by immunoblot with anti-mRFP antibody. +: CsA (20 μg/ml), -: DMSO. File for the primary data corresponding to Figure 4—figure supplement 1G. [file elife-79179-fig4-figsupp1-data2.zip › Original image-anti-Ub.jpg]

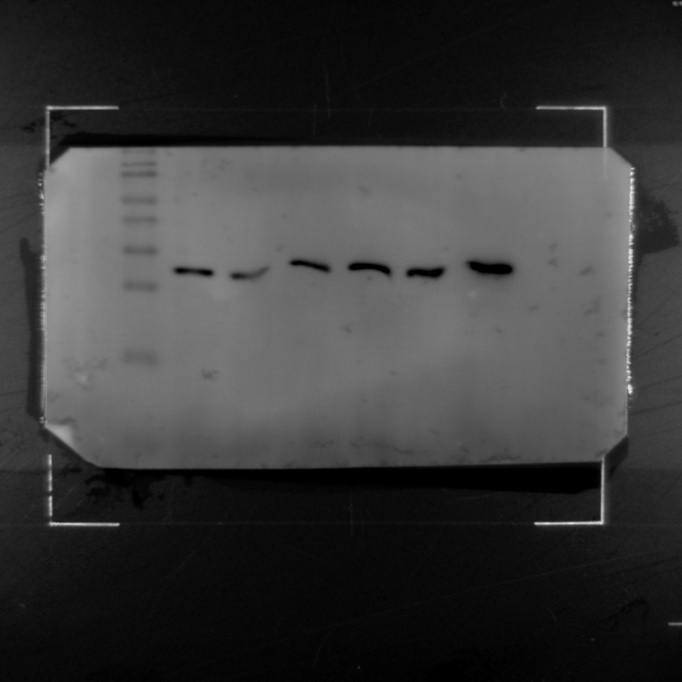

Supplement: Figure 4—figure supplement 1—source data 2. — Uncropped western blot is to demonstrate K264 in N terminus is responsible for the ubiquitination of BN258-268 (K264A)::mRFP. Hyphae were incubated with anti-mRFP affinity beads 4FF. IP materials (anti-mRFP) were subjected to immunoblot with anti-Ub (ubiquitin) and anti-mRFP antibodies. The total extracts (input) were detected by immunoblot with anti-mRFP antibody. +: CsA (20 μg/ml), -: DMSO. File for the primary data corresponding to Figure 4—figure supplement 1G. [file elife-79179-fig4-figsupp1-data2.zip › Original image-Anti-mRFP-IP.jpg]

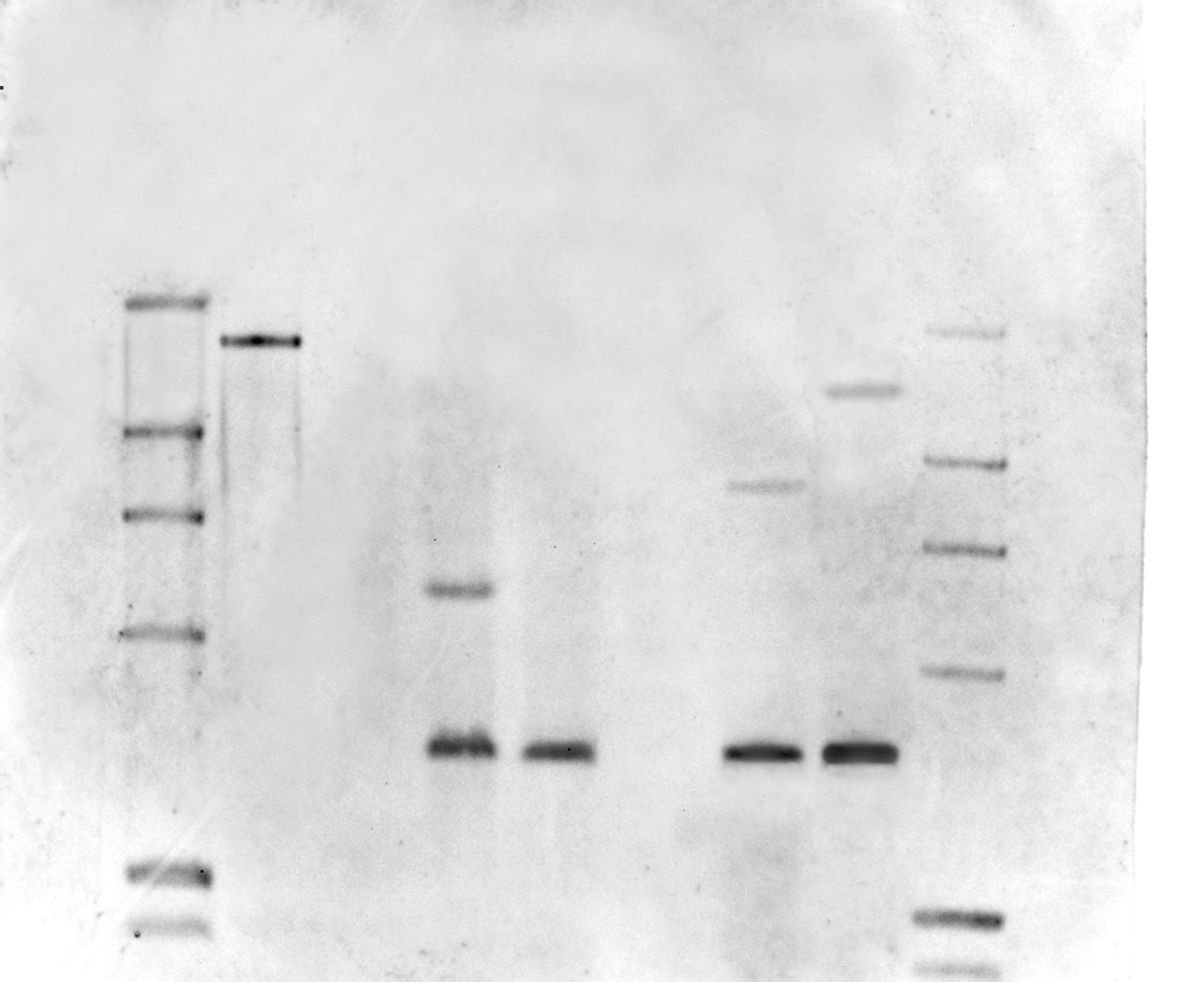

Supplement: Figure 5—figure supplement 1—source data 2. — File for the primary data corresponding to Figure 5—figure supplement 1A, C and E . [file elife-79179-fig5-figsupp1-data2.zip › Original image-Southern blot.jpg]

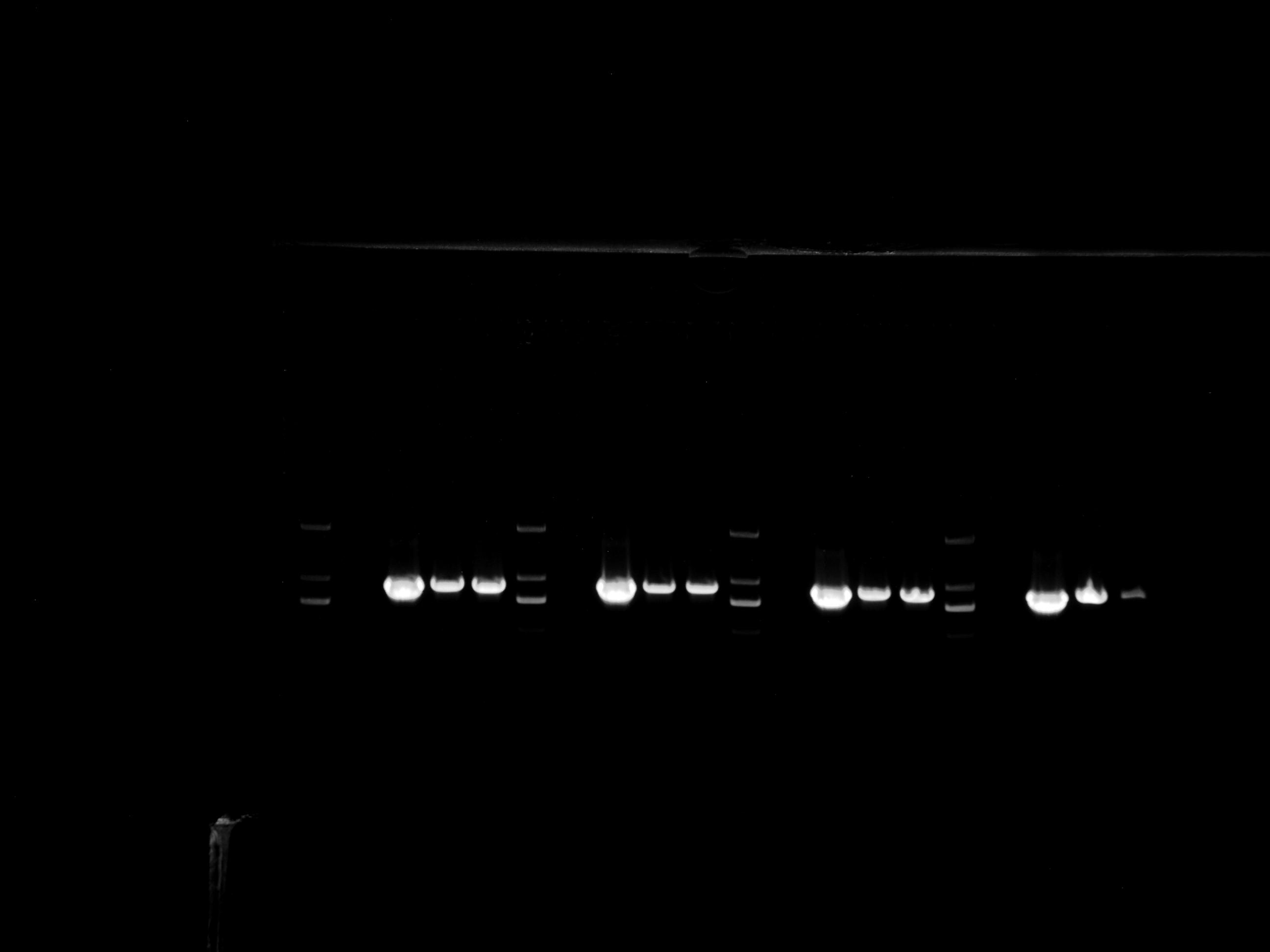

Supplement: Figure 5—figure supplement 1—source data 2. — File for the primary data corresponding to Figure 5—figure supplement 1A, C and E . [file elife-79179-fig5-figsupp1-data2.zip › Original image-PCR validation of transgenic plants.jpg]
