# Supplementary material for: Cell detoxification of secondary metabolites by P4-ATPase-mediated vesicle transport
Source: eLife. 2023 Jul 4;12:e79179. doi: 10.7554/eLife.79179 (PMC10322151; doi:10.7554/eLife.79179)
Supplement: Figure 4—figure supplement 1—source data 2. — Uncropped western blot is to demonstrate K264 in N terminus is responsible for the ubiquitination of BN258-268 (K264A)::mRFP. Hyphae were incubated with anti-mRFP affinity beads 4FF. IP materials (anti-mRFP) were subjected to immunoblot with anti-Ub (ubiquitin) and anti-mRFP antibodies. The total extracts (input) were detected by immunoblot with anti-mRFP antibody. +: CsA (20 μg/ml), -: DMSO. File for the primary data corresponding to Figure 4—figure supplement 1G. [file elife-79179-fig4-figsupp1-data2.zip › Ubiquitination detection.pptx]

## Slide 1
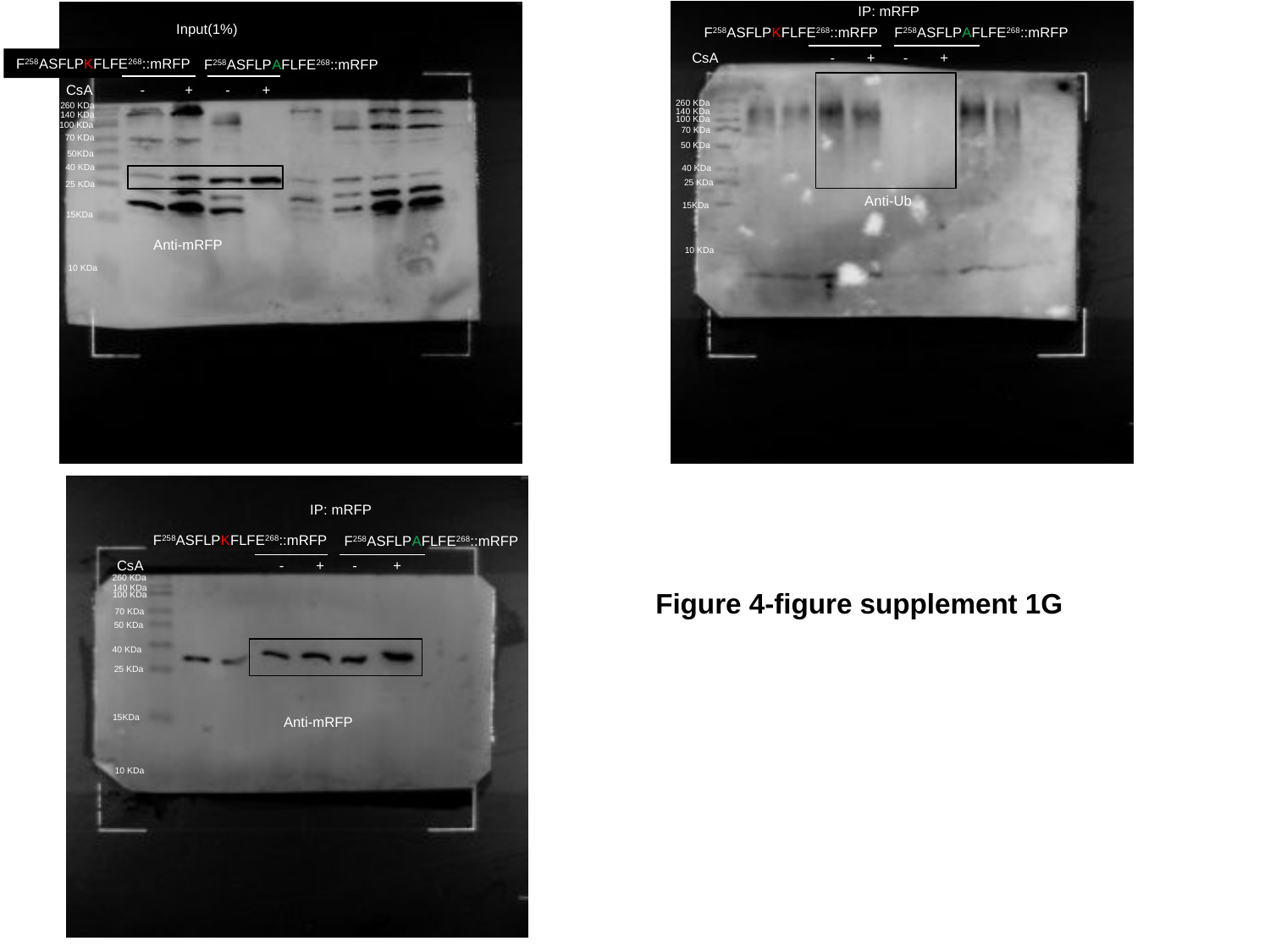

IP: mRFP
F258ASFLPKFLFE268::mRFP
F258ASFLPAFLFE268::mRFP
 CsA - + - +
Anti-Ub
100 KDa
70 KDa
50 KDa
40 KDa
25 KDa
15KDa
10 KDa
Input(1%)
F258ASFLPKFLFE268::mRFP
F258ASFLPAFLFE268::mRFP
CsA - + - +
Anti-mRFP
100 KDa
70 KDa
 50KDa
40 KDa
25 KDa
15KDa
10 KDa
260 KDa
260 KDa
140 KDa
140 KDa
IP: mRFP
F258ASFLPKFLFE268::mRFP
F258ASFLPAFLFE268::mRFP
 CsA - + - +
Anti-mRFP
100 KDa
70 KDa
50 KDa
40 KDa
25 KDa
15KDa
10 KDa
260 KDa
140 KDa
Figure 4-figure supplement 1G
