# Supplementary material for: Cell detoxification of secondary metabolites by P4-ATPase-mediated vesicle transport
Source: eLife. 2023 Jul 4;12:e79179. doi: 10.7554/eLife.79179 (PMC10322151; doi:10.7554/eLife.79179)
Supplement: Figure 5—figure supplement 1—source data 2. — File for the primary data corresponding to Figure 5—figure supplement 1A, C and E . [file elife-79179-fig5-figsupp1-data2.zip › Validation of transgenic BbCRPA plants.pptx]

## Slide 1
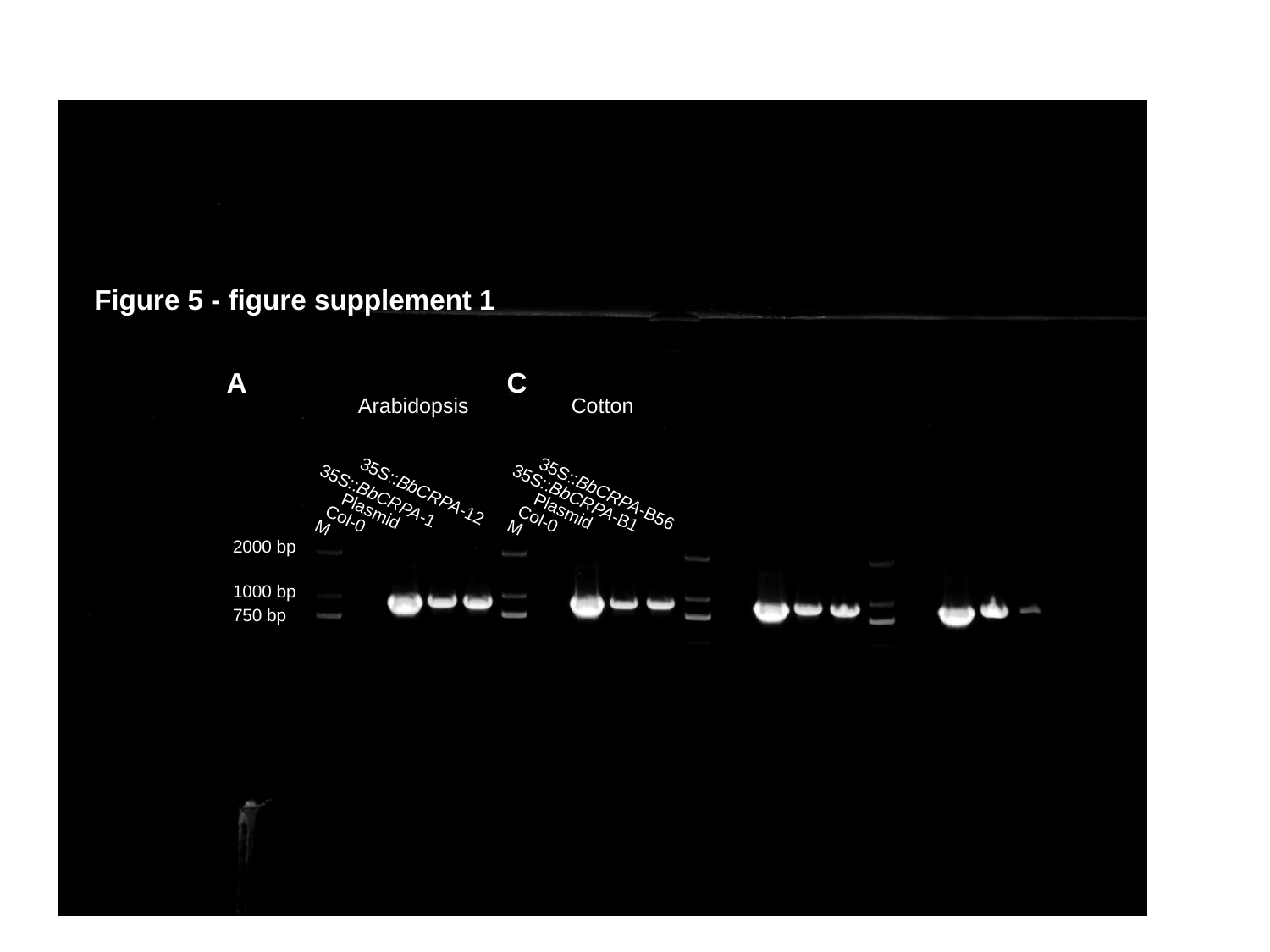

Figure 5 - figure supplement 1
A
C
Arabidopsis
Cotton
35S::BbCRPA-12
35S::BbCRPA-B56
35S::BbCRPA-1
35S::BbCRPA-B1
Plasmid
Plasmid
Col-0
Col-0
M
M
2000 bp
1000 bp
750 bp

## Slide 2
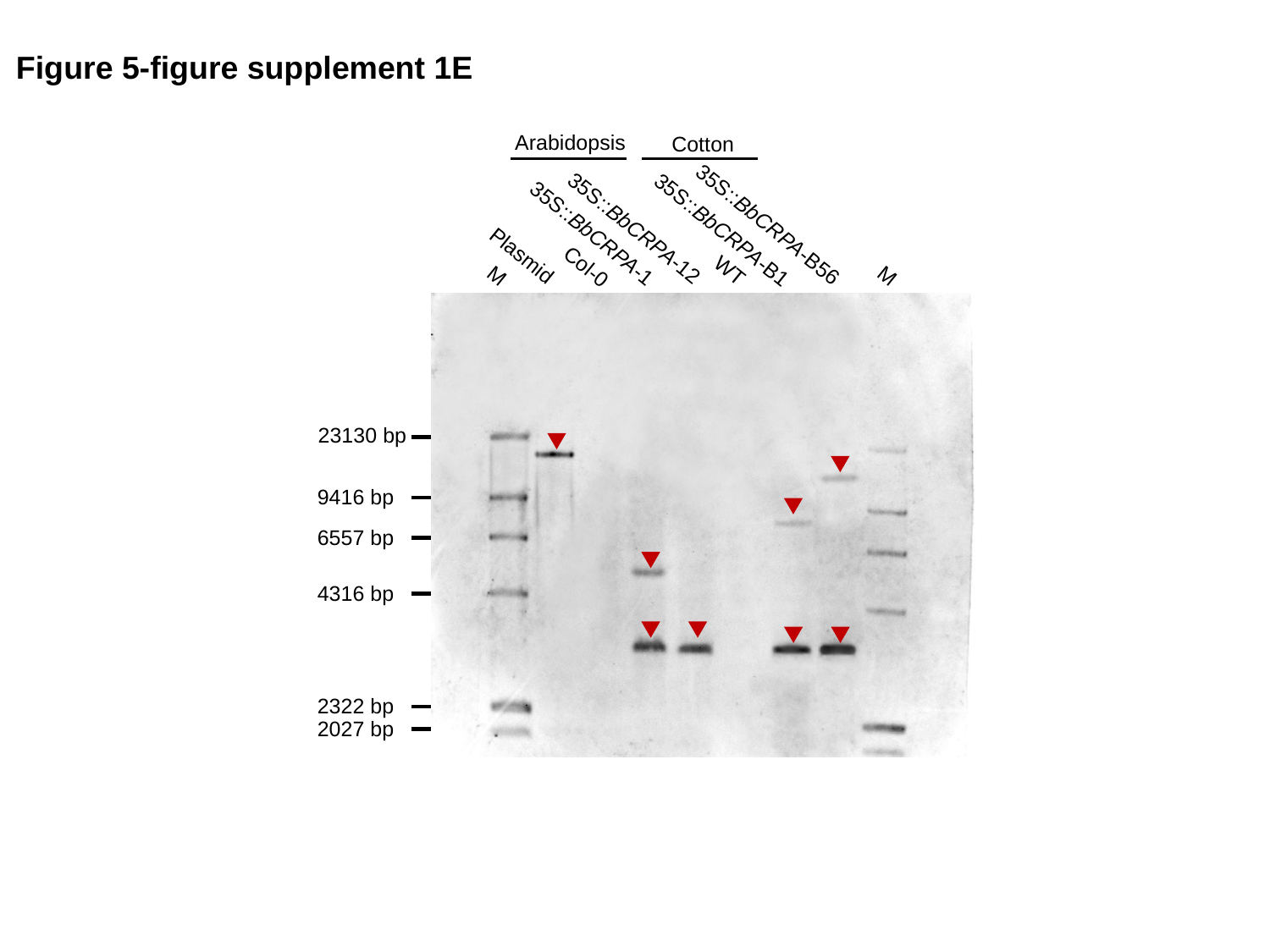

Figure 5-figure supplement 1E
Arabidopsis
Cotton
35S::BbCRPA-B56
35S::BbCRPA-12
35S::BbCRPA-B1
35S::BbCRPA-1
Plasmid
M
M
Col-0
WT
23130 bp
9416 bp
6557 bp
4316 bp
2322 bp
2027 bp
