# Supplementary material for: Cell detoxification of secondary metabolites by P4-ATPase-mediated vesicle transport
Source: eLife. 2023 Jul 4;12:e79179. doi: 10.7554/eLife.79179 (PMC10322151; doi:10.7554/eLife.79179)
Supplement: Supplementary file 1. [file elife-79179-supp1.docx]

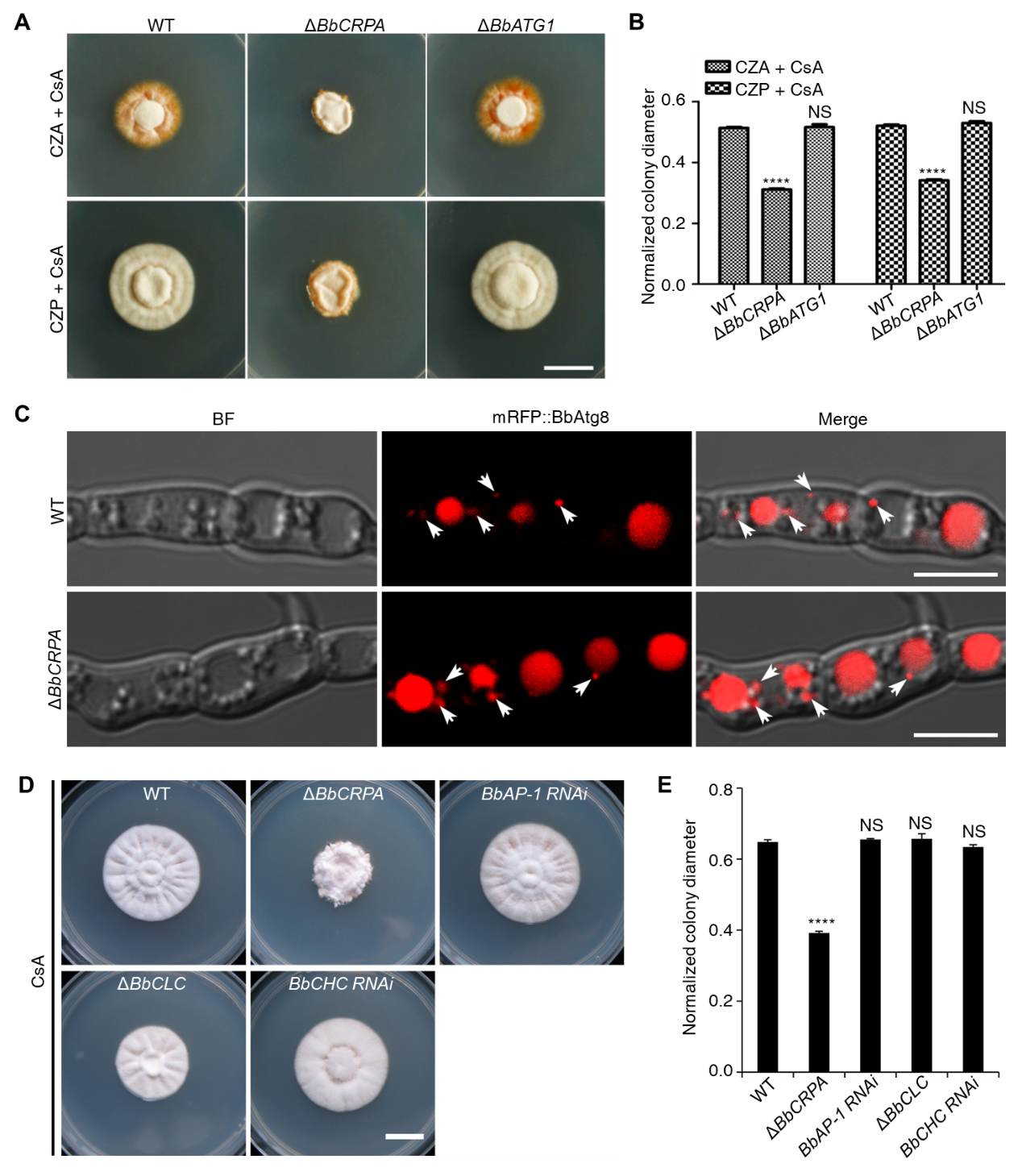
**Supplementary file 1.** Loss of autophagy-related protein BbAtg1, adaptor protein BbAP-1, or coat protein clathrin does not affect CsA resistance. **(A and B)** Growth of the wild-type, Δ*BbCRPA*, and Δ*BbATG1* strains on CZA/CZP + CsA (20 μg/ml). **(C)** Disruption of *BbCRPA* does not affect the autophagosome formation in *B. bassiana.* Autophagosomes are marked by mRFP::BbAtg8 (arrows). **(D and E)** Growth of the wild-type, Δ*BbCRPA*, *BbAP-1 RNAi*, Δ*BbCLC*, and *BbCHC* strains on CZP + CsA (20 μg/ml). CLC, clathrin light chain; CHC, clathrin heavy chain. For CsA sensitivity analysis, plates were spot inoculated with 3 μl conidial suspensions (1 × 10^7^ conidia/ml) and incubated at 26℃ for about 10 days. Different strains showed variation in growth rates and the data were shown as [colony diameter CZA/CZP supplemented with CsA ]/[colony diameter CZA/CZP]. Scale bars, 1 cm for (**A and D**) and 5 μm for (**C**). Data are represented as mean ± SD. ****p < 0.0001 from Student’s *t* test. NS, not signiﬁcant.
