# Supplementary material for: Cell detoxification of secondary metabolites by P4-ATPase-mediated vesicle transport
Source: eLife. 2023 Jul 4;12:e79179. doi: 10.7554/eLife.79179 (PMC10322151; doi:10.7554/eLife.79179)
Supplement: Supplementary file 2. [file elife-79179-supp2.docx]

**Primers used in this study**

| **Primer name Sequence (5’ to 3’)** | **Restriction enzyme sites^a^ Comment** |
| --- | --- |
| **Construction and validation of *BbCRPA* gene knock-out and complemented strains**  *BbCRPA*LB-F cgGAATTCcgCGATGTCGTTCGAGTCGAAT  *BbCRPA*LB-R cgGAATTCcgGTGTAGGCGTTCCAGGATGA  *BbCRPA*RB-F gcTCTAGAgcAGCTTGCCATAATGTGCAAG  *BbCRPA*RB-R cccAAGCTTgggTTCTTGGTGGTGTCGTAGGC  Com-F gACTAGTGGGATGACCCGGAACTCCTGAGAA  Com-R gcTCTAGACTCACCAACACATGAAGGCG  MCS-F CGTAAGAGAATGTCGACCAT  MCS-R CTGAATCATCGACACGTCGT  *Sur*-F aaggaaaaaaGCGGCCGCGACGACTGAGAACAGATTCG  *Sur*-R cgGGATCCGTCGACGTGAGAGCATGCAATTC    **Construction of tagged fusion proteins**  *BbCRPAN***-**F aaggaaaaaaGCGGCCGCaaggaaaaaaATGGC  TGGACGACCTCCTGG  *BbCRPA* (*mF*)-F GGTGGTTCTGGTGGTGGTTCTGGTATGGCTGGACGA  CCTCCTGG  *BbCRPAN*-R cgGGATCCCTATTGCGGACGCGAGCTGG  *eGFPN*-F aaggaaaaaaGCGGCCGCGTCGCCACCATGGTGAGCAA  *eGFPN*-R aaggaaaaaaGCGGCCGCACCAGAACCACCACCAGAAC  CACCGGACTTGTACAGCTCGTCCA  *eGFP*(*NF*)-R ACCAGAACCACCACCAGAACCACCGGACTTGTACAGCTC  GTCCA  *BbRab5*-F GGTGGTTCTGGTGGTGGTTCTGGTATGGCCTCCCGAGG  ACCCCCT  *BbRab5*-R cgGGATCCTTAACAGCTGCAAGGGCCAG  *BbRab7*-F GGTGGTTCTGGTGGTGGTTCTGGTATGTCTTCACGCAAGAAGGT  *BbRab7*-R cgGGATCCTTAACAGGCACAGCCGTCGC  *mRFP*N-F aaggaaaaaaGCGGCCGCATGGCCTCCTCCGAGGACGT  *mRFP*N-R ACCAGAACCACCACCAGAACCACCGGCGCCGGTGGAGTGGCG  GCCCTCGGCGCGCTCGTACTGTT  *P1*-F gcTCTAGAGTTGGGTATGCTCCGGCGCG  *T1*-R gcTCTAGAAAGAAGGATTACCTCTAAAC  *mPH*-F gcTCTAGATCGACCCATCCGGTGCTCTG  *mPH-*R gcTCTAGAACTAGTCAATAGTGGTGAAA  *DRS2* (*Fu*)-F GGTGGTTCTGGTGGTGGTTCTGGTATGAATGACGA  CAGAGAAAC  *DRS2*-R aaggaaaaaaGCGGCCGCTCATATATCAAATGAAATATCATCTCTCG  *P2*-F gACTAGTGTTGGGTATGCTCCGGCGCG  *T2*-R gACTAGTAAGAAGGATTACCTCTAAAC  *mRFP*-F GTTTTTACCAAAGTTTCTTTTCGAGATGGCCTCCTCCGA  GGACGT  *mRFP*-R cgGGATCCTTAGGCGCCGGTGGAGTGGC  *N268*-R TGATGACGTCCTCGGAGGAGGCCATCTCGAAAAGA  AACTTTGGTA  *N258-268*-F aaggaaaaaaGCGGCCGCATGTTTGCATCGTTTTTACCAAAG  TTTCTTTTCGAGATGGCCTCCTCCGAGGACGT  *N258-268* (*K-A*)-F aaggaaaaaaGCGGCCGCATGTTTGCATCGTTTTTACCAGC  *N259-268*-F aaggaaaaaaGCGGCCGCATGGCATCGTTTTTACCAAAG  TTTCTTTTCGAGATGGCCTCCTCCGAGGACGT  **Site-directed mutagenesis of *BbCRPA***  *BbCRPA*-F aaggaaaaaaGCGGCCGCATGGCTGGACGACCTCCTGG *Bb* (*D614R*)-first-R AGTGAGAGTACCAGTCTTGCGAGAAAAGACATA  CTCG ACCATAC  *Bb* (*D614R*)-second-F TCTCGCAAGACTGGTACTCTCACTTGCAACATGA  TGGA GTTCAA  *Bb* (*I562E*)-first-R GTTCAACAGTGACAAATAGTGACTCGGGAACGAG  GGCAGAGAACA  *Bb* (*I562E*)-second-F GGTCCTGTTCTCTGCCCTCGTTCCCGAGTCACTA  TTTGTCACTGT  *Bb* (Δ*C1325-1359*)-R cgGGATCCCTAGTAGCCGCGCTGCTTTCGCAT  *Bb* (Δ*C1326-1359*)-R cgGGATCCCTAGCCGCGCTGCTTTCGCATGC  *Bb* (Δ*N*)-F aaggaaaaaaGCGGCCGCATGCAATTCTCCAAAGTTGCCAA  *Bb* (Δ*C*)-R cgGGATCCCTACCACGCAAAGTCGCGGAGCA  *Bb* (*1325*)-R cgGGATCCCTATTGCGGACGCGAGCTGGCCATTTCACCGTA  CCGTCCACGGTTCTTGGTGGTGTCGTAGGCCTGCAGCACGC  GGGTCTGGCTCTCGTCGGCCTGGGAAAAGGCTGCGCCGCGCTGCTTTCGCATGC  *Bb* (*1325*-*1341*)-R cgGGATCCCTATTGCGGACGCGAGCTGGCCATTTCACCG  TACCGTCCACGGTTCTTGGTGGTGTCTGCGGCCTGCAGCACGCGGGTCTGGCTCTCGTCGGCCTGGGAAAAGGCTGCGCCGCGCTGCTTTCGCATGC  *Bb* (*1325*-*1350*)-R cgGGATCCCTATTGCGGACGCGAGCTGGCCATTTCACCT  GCCCGTCCACGGTTCTTGGTGGTGTCGTAGGCCTGCAGCACGCGGGTCTGGCTCTCGTCGGCCTGGGAAAAGGCTGCGCCGCGCTGCTTTCGCATGC  *Bb* (*1341*-*1350*)-R cgGGATCCCTATTGCGGACGCGAGCTGGCCATTTCACCTG  CCCGTCCACGGTTCTTGGTGGTGTCTGCGGCCTGCAGCAC  GCGGGTCT  *Bb*(*1325*-*1341*-*1350*)-R cgGGATCCCTATTGCGGACGCGAGCTGGCCATTT  CACCTGCCCGTCCACGGTTCTTGGTGGTGTCTGCGGCCTGCAGCACGCGGGTCTGGCTCTCGTCGGCCTGGGAAAAGGCTGCGCCGCGCTGCTTTCGCATGC  **N/C-terminal tail exchange between BbCrpa and Drs2p**  *Bb*N*-*R ACCAATTGTGGTGTATCTATTAGTCTCGAAAAGAAAC  TTTGGTA  *DRS2TMD*-F TTTTTACCAAAGTTTCTTTTCGAGACTAATAGATACACCA  CAAT  *DRS2TMD*-R GTCGGTACATGCGCTTGGCATACTTTACCAGTGCAA  AAATT GGTA  *DRS2*-F aaggaaaaaaGCGGCCGC ATGAATGACGACAGAGAAAC  *BbC* -F CGTTTTACCAATTTTTGCACTGGTAAAGTATGCCAA  GCGCATGTA  *BbCRPA-R* aaggaaaaaaGCGGCCGCCTATTGCGGACGCGAGCTGG  *DRS2N* -R GACATTGGCAACTTTGGAGAATTGTGGCGAGACGTGAG  GCACCT  *BbTMD*-F CAACAGGTGCCTCACGTCTCGCCACAATTCTCCAAAGT  TGCCAA  *BbTMD*-R TATAGTACTTCCATAGAAAATCTCTCCACGCAAAGTCGC  GGAGCA  *DRS2*C-F CTGCCTGCTCCGCGACTTTGCGTGGAGAGATTTTCTATG  GAAGTA  **Gene** **expression analyses**  *BbCRPA*ex-F CTGGAGGAGTATGCTTCCGA  *BbCRPA*ex-R TATCACCAGTGAGGACCCAG  *BbCRPA* (plant)-F AACAGGCCGATAATGCGCTA  *BbCRPA* (plant)-R GTCTCGGGAAGTCCTTGCTT  *actin*-F TTGGTGCGAAACTTCAGCGTCTAGTC  *actin*-R TCCAGCAAATGTGGATCTCCAAGCAG  *AtActin*2-F GATTCAGATGCCCAGAAAGTCTTG  *AtActin*2-R TGGATTCCAGCAGCTTCCAT  *GhHis3*-F CCGTCCTGGAACTGTTGCTCT  *GhHis3*-R ACCCACAAGGTATGCCTCTGC  **Plant transformation**  *pBbCRPA*-F cgGGATCCcgATGGCTGGACGACCTCCTGG  *pBbCRPA*-R ggACTAGTccCTATTGCGGACGCGAGCTGG  **Southern blot**  *BbCRPA*-F1 GCGCAATGTACAGCTACAC  *BbCRPA*-R1 AGGCGTTCAAGAATGACGGT | *Eco*RI Cloning of 5'-end of *BbCRPA*  *Eco*RI  *Xba*I Cloning of 3'-end of *BbCRPA*  *Hin*dIII  *Spe*I *BbCRPA* complemented strain construction  *Xba*I  *BbCRPA* mutant/complemented strain screening  *Not*I Cloning of *Sur* cassette  *Bam*HI  *Not*I Cloning *BbCRPA* ORF      *Bam*HI  *Not*I Cloning of *eGFP*  *Not*I      Cloning of *BbRab5*  *Bam*HI  Cloning of *BbRab7*  *Bam*HI  *Not*I Cloning of *mRFP*    *Xba*I Cloning of *mRFP*::*Rab7* cassette  *Xba*I  *Xba*I Cloning of *mRFP*::*PH^OSBP^* cassette  *Xba*I  Cloning of *DRS2* ORF    *Not*I  *Spe*I Cloning of *eGFP*::*DRS2* cassette  *Spe*I  Cloning of *mRFP*    *Bam*HI  Cloning of part of BbCrpa N-terminus    *Not*I Cloning of part of BbCrpa N-terminus    *Not*I Cloning of part of BbCrpa N-terminus  *Not*I Cloning of part of BbCrpa N-terminus  *Not*I Cloning part of *BbCRPA* ORF    *Bam*HI  *Bam*HI  *Not*I  *Bam*HI  *Bam*HI  *Bam*HI        *Bam*HI  *Bam*HI    *Bam*HI      Cloning N-terminus of BbCrpa  Cloning the transmembrane domains of Drs2p  *Not*I Cloning of *DRS2* ORF  Cloning C-terminus of BbCrpa  *Not*I Cloning of *BbCRPA* ORF  Cloning N-terminus of Drs2p    Cloning of the transmembrane domains of BbCrpa  Cloning C-terminus of Drs2p    qRT-PCR analysis of *BbCRPA for B. bassiana*  qRT-PCR analysis of *BbCRPA* for plants  Internal reference in qRT-PCR analysis  *Bam*HI Cloning of *BbCRPA* ORF  *Spe*I  Cloning of probe for southern blot |

**^a^Underlined sequences are restriction enzyme sites and lowercase letters denote protective bases.**
